# Supplementary material for: Multi-objective optimization of wear parameters of hybrid composites (LM6/B 4 C/fly ash) using grey relational analysis
Source: PLoS One. 2025 Jun 26;20(6):e0326086. doi: 10.1371/journal.pone.0326086 (PMC12200873; doi:10.1371/journal.pone.0326086)
Supplement: Attachment — Submitted filename: Reviewer comments_Plos One.docx [file pone.0326086.s001.docx]

Reviewer Comments

1. The manuscript fails to clearly establish the novelty of the study. Combining GRA with wear analysis is not a new approach, and the authors do not explain how their work differs from or advances previous studies.
2. The choice of LM6 as the matrix material and B4C and fly ash as reinforcements is not adequately justified. The manuscript lacks discussion on why these materials were chosen, particularly regarding their practical or industrial significance.
3. The methodology section lacks critical details about the stir casting process. Key parameters such as stirring speed, temperature control, and reinforcement feeding methods are missing, making the process non-reproducible.
4. The particle size, morphology, and distribution of B4C and fly ash were not characterized or reported. This raises concerns about the homogeneity of the composite.
5. The rationale behind the selection of specific wear test parameters (load, speed, and sliding distance) and their ranges is not provided. The relevance of these ranges to real-world conditions is unclear.
6. ANOVA results are presented but not adequately interpreted. The manuscript does not discuss whether the assumptions of ANOVA (e.g., normality, homoscedasticity) were validated.
7. The discussion section merely restates the results without providing a mechanistic explanation. For example, the effect of reinforcement on wear resistance is discussed without considering particle-matrix interactions, interfacial bonding, or load transfer mechanisms.
8. The influence of particle size and distribution on wear behaviour is completely ignored.
9. Figures, such as micrographs, lack adequate descriptions. There is no explanation of how the microstructural features relate to wear performance.
10. Tables lack clear labelling, and some critical information (e.g., units for certain variables) is missing. For example, in tables showing wear rate, the units are not explicitly stated.
11. Some references cited are outdated or irrelevant. Recent studies in wear behaviour of hybrid composites should be included.
12. The abstract and conclusion sections are repetitive and lack focus. They fail to highlight specific quantitative results or the practical implications of the findings.
13. The manuscript contains numerous grammatical errors and awkward phrasing, which hinder readability. For example, sentences are overly long and contain redundant information.
14. Equations and their variables are not consistently formatted or explained in the text.
15. While GRA is used for optimization, there is no cross-validation or verification of the results. Without experimental validation of the optimal parameter combination, the reliability of the optimization is questionable.

**Recommendation:**

**Rejected**

The manuscript lacks sufficient quality in terms of novelty, methodological rigor, and scientific depth. Significant improvements are needed in experimental design, data analysis, and presentation to meet the standards of a peer-reviewed journal. If resubmitted, the authors must address the issues listed above comprehensively.
